# The Different Paths That Lead to Hypotonic Hyponatremia, and a Safe Approach to Treatment

**DOI:** 10.3390/jcm14010092

**Published:** 2024-12-27

**Authors:** Louis J. Imbriano, Candace Grant, Naveed Masani

**Affiliations:** NYU Grossman Long Island School of Medicine, 101 Mineola Blvd., Mineola, NY 11501, USA; candace.grant@nyulangone.org (C.G.); naveed.masani@nyulangone.org (N.M.)

**Keywords:** syndrome of inappropriate antidiuretic hormone, inflammation, acute phase reactants, urate, haptoglobin-related protein

## Abstract

A knowledge gap may exist when attempting to identify the pathogenetic mechanisms resulting in the syndrome of inappropriate antidiuretic hormone (SIADH) or hypotonic hyponatremia. Ectopic secretion of antidiuretic hormone [ADH] is the classic cause of SIADH. But another form of inappropriate secretion of ADH occurs when interleukin 6 is activated. Hypotonic hyponatremia can also occur in patients with cerebral salt wasting, but the secretion of ADH is appropriate, responding to volume depletion induced by excessive natriuresis. Reset osmostat (RO) is another cause of hypotonic hyponatremia caused by an unknown anomaly in the hypothalamus. This review discusses the pathophysiology of and the identical laboratory findings found in classic ectopic ADH secretion, interleukin 6-mediated ADH secretion, cerebral salt wasting-induced ADH secretion, and RO. This review also discusses potential methods to discern which hypotonic hyponatremic syndrome is present and current recommendations for treatment.

## 1. Introduction

This review describes the different causes of hypotonic hyponatremia that are referred to as the syndrome of inappropriate antidiuretic hormone (SIADH). The first, classic, description of SIADH referred to ectopic, unregulated secretion of ADH by a tumor. Subsequently, a list of criteria and features of the laboratory presentation were described for the diagnosis of SIADH. However, recent studies have shed light on two other mechanisms causing excessive ADH production which also present the same laboratory presentation. Interleukin 6 (IL-6)-mediated ADH secretion is also inappropriate, occurring during the course of inflammation, while another syndrome referred to as cerebral salt wasting (CSW) is an appropriate volume-mediated ADH secretion. Cerebral salt wasting syndrome is the most frequent cause of hyponatremia in patients with subarachnoid hemorrhage [1], but salt wasting has been described in other instances, and perhaps the term “cerebral” should be changed to “cerebral–renal salt wasting” [2]. There is no controversy regarding the existence of CSW, since treatment guidelines already incorporate recommendations “regardless of the volume status” and warn that water restriction or the use of tolvaptan should be avoided.

Classic SIADH and IL-6-mediated SIADH patients are euvolemic, but CSW patients are hypovolemic due to excessive natriuresis. The three syndromes present with hypotonic hyponatremia and the same laboratory profile, which creates a clinical dilemma regarding diagnosis and treatment.

## 2. Hypotonic Hyponatremia

Hyponatremia is defined as a serum sodium value < 135 mEq/L. It is the most common electrolyte disturbance, with a prevalence of up to 35% of hospitalized patients and up to 7% of ambulatory patients [3]. Mohan et al. estimated the prevalence of hyponatremia in the United States as 1.72%, higher in females, increasing with age, and more common in subjects with hypertension, diabetes, coronary artery disease, stroke, chronic lung disease, cancer, or psychiatric disorders. In their study there was a significant risk of death, with a U-shaped relationship between serum sodium and hazard ratio for mortality [4].

The symptoms of hyponatremia range from subtle neurological anomalies affecting gait to frequent falls, writing difficulty, headaches, confusion, seizures, coma, or death. The symptoms vary depending on the level of serum sodium and the rate of fall of serum sodium.

SIADH was first described in 1957. Two patients with lung cancer had hyponatremia, and it was believed that the tumor was an ectopic source of ADH [5]. Ectopic production of ADH should retain the original designation of “classic SIADH”. In this “classic” description of SIADH, the secretion of ADH was not stimulated by high plasma osmolality, low plasma volume, or hypotension, which are the normal triggers to activate the hypothalamus and pituitary. Instead, in “classic SIADH” the secretion of ADH is ectopic, unsuppressed, unregulated, and autonomous by a tumor. The excess antidiuretic hormone stimulates reabsorption of water by the kidneys, resulting in an increase in the volume of extracellular fluid (ECF) and a decrease in serum sodium. The Schwartz et al. criteria for SIADH include serum sodium (SNa) < 135 mOsm/L, plasma osmolality (Posm) < 275 mOsm/L, urine osmolality (Uosm) > 100 mOsm/L, urinary sodium (Una) > 40 mmol/L, euvolemia, and normal renal, adrenal, thyroid function, as well as the absence of cardiac failure, pituitary insufficiency, hepatic disease, or recent diuretic use [6]. Since 1957, many cases of hyponatremia that appear to meet these criteria have been ascribed to SIADH. Online searches for causes of “SIADH” show many unrelated disorders (Table 1).

Table 1 includes the nonosmotic stimuli of ADH secretion by drugs, hormone deficiencies, and hereditary hyponatremia associated with upregulation of the V2 receptor, as well as the classic ectopic secretion of ADH. However, many of the other causes of SIADH defy a unifying pathogenesis; but it is becoming more evident every year that inflammation is a common underlying theme. The diversity of syndromes, including infections, autoimmune disorders, trauma, lung disorders, and intracranial bleeding, are not due to ectopic ADH secretion but rather to inflammation-mediated ADH secretion. The frequency of inflammation with increased levels of ADH may explain why hyponatremia is so ubiquitous and included in lists of SIADH.

**Figure 1:**
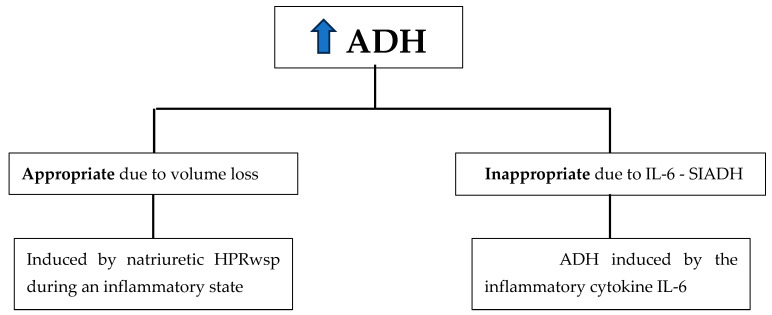
Inflammation-induced ADH secretion, by different mechanisms.

Inflammation-induced hypotonic hyponatremia syndromes may be caused by inappropriately induced ADH secretion by IL-6, or by appropriately induced ADH secretion in response to volume depletion caused by an inflammation-induced natriuretic protein. One mechanism involves IL-6, which inappropriately induces ADH secretion during inflammatory states. The other mechanism involves a different inflammatory protein, haptoglobin-related protein without signal peptide (HPRwsp), which causes renal salt wasting, volume depletion, and appropriate stimulation of ADH secretion [7]. This is a current hypothesis which deserves increased investigation. The secretion of ADH is not ectopic but inappropriate in patients with IL-6 being the direct inducer of ADH, while the secretion of ADH is appropriate in CSW patients, with HPRwsp being the predominant acute phase reactant. These two distinct inflammation-induced hypotonic hyponatremia syndromes, as well as cases of classic ectopic ADH production, present with remarkably similar laboratory findings, resulting in diagnostic confusion, and should be considered as three distinct entities. Each of the three presentations may require urgent treatment, which can differ depending on which mechanism is present (Figure 1). Because the treatment of CSW requires volume repletion, while the treatment of classic SIADH and IL-6-mediated SIADH requires free water excretion via salt and protein intake, a critical management must be made. This realization has led to a broad-based treatment recommendations for hypoosmotic hyponatremia that have excluded fluid restriction.

## 3. Inflammation Induces Cytokines

**Figure 2:**
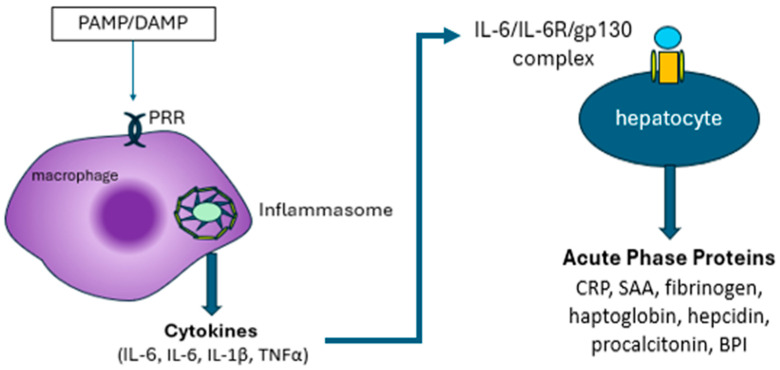
Events from recognition of “antigens” to release of acute phase reactants.

At sites of inflammation, microbial molecules known as pathogen-associated molecular patterns (PAMPs), as well as native damage-associated molecular patterns (DAMPs), are detected by pattern recognition receptors (PRRs) and Toll-like receptors (TLRs) on innate immune cells such as macrophages and monocytes. Intracellular signaling induces cytosolic inflammasomes, multimeric protein complexes, to direct the macrophages and monocytes (as well as B-lymphocytes, T-lymphocytes, endothelial cells, fibroblasts, and mast cells) to produce various cytokines, including interleukins such as IL-6, as well as interleukin 1β (IL-1β) and tumor necrosis factor-α (TNFα). IL-6 circulates and couples with cell surface receptors (IL-6Rs) to induce the liver to produce acute phase proteins such as C-reactive protein (CRP), serum amyloid A (SAA), fibrinogen, haptoglobin (3 phenotypes and two variants), hepcidin, procalcitonin, bactericidal permeability increasing protein (BPI), and others (Figure 2).

Circulating IL-6 demonstrates pleiotropic effects on inflammation, cellular immune responses, cell-to-cell signaling, stimulation of the acute phase response (originally known as hepatocyte-stimulating factor), anti-viral effects (originally known as interferon-β2), defense of neurons, neuronal differentiation, and survival of B-cells (originally known as B-cell stimulatory factor-2). IL-6 also inhibits and limits the proinflammatory activity of IL-1β and TNFα, restricting inflammatory warfare [8]. Importantly, IL-6 also induces the nonosmotic, inappropriate secretion of ADH [9,10].

## 4. The Role of Inflammation-Induced IL-6 in Hyponatremia

Studies by Spath-Schwalbe et al. have suggested that interleukin 6 stimulates the secretory activity of the hypothalamus–pituitary–adrenocortical axis (HPA), with rapid increases in plasma adrenocorticotropic hormone (ACTH) and cortisol levels after injection of IL-6 [11]. A similar study by Mastorakos et al. showed that injected IL-6 induced ACTH, cortisol, and ADH, suggesting that IL-6 activated the magnocellular AVP-secreting neurons and might be involved in SIADH [12]. Melmed has referred to this phenomenon as “the immune-neuroendocrine interface” [13].

Circulating IL-6 couples with IL-6 receptor (IL-6R) subunits on cell surfaces and then, via a signal-transducing subunit (gp130), leads to transcription of target genes. Based on the fact that IL-6Rs are present in the supraoptic (SON) and paraventricular nuclei (PVR), as well as the subfornical organ (SFO) and the organum vasculosum of the lamina terminalis (OVLT), some authors have speculated that IL-6 could directly stimulate the secretion of vasopressin. Alternatively, some research has shown that endothelial cells, smooth muscle cells, and pericytes of the blood brain barrier secrete IL-6 in response to IL-1β and even lipopolysaccharide (LPS), representing a direct, local induction of vasopressin by LPS [14,15,16]. LPS-induced vasopressin release was shown to be independent of known stimuli such as serum osmolality, hypotension, or volume depletion [17].

Many studies have demonstrated that IL-6-mediated hyponatremia is associated with a variety inflammatory conditions including head injuries, bodily injury in traffic accidents, and autoimmune diseases, as well as in prolonged exercise [18,19,20,21,22].

**Figure 3:**
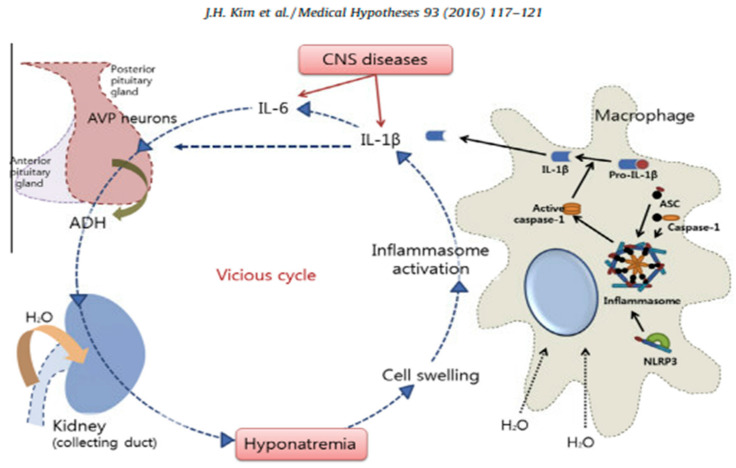
Amplification of hyponatremia by cellular hypotonicity [23].

The severity of hyponatremia induced by inflammation may be further amplified as cells become hypo-osmolar. Intracellular hypo-osmolality further activates the inflammasome with increased cytokine production of IL-1β and IL-6, contributing to a vicious cycle of more ADH and hyponatremia [23]. Amplification of the immune-neuroendocrine interface is depicted in Figure 3.

Two unanswered questions regarding the “neuro-biological pathways” include why some infections cause hyponatremia more often than others and if vasopressin can also be produced ectopically during infection [24].

## 5. The Role of Inflammation and Haptoglobins in Inducing Hyponatremia in Cerebral Salt Wasting

Haptoglobin (Hp) is a liver-derived glycoprotein, one of several acute phase reactants induced by IL-6, whose serum levels rise several fold during inflammation. Three main Hp phenotypes exist––Hp1-1, Hp1-2, and Hp2-2––as well as a variant known as haptoglobin-related protein (Hpr) [25]. All of the Hp phenotypes have differing biologic activity, both pro-inflammatory and anti-inflammatory [26], such as binding toxic free hemoglobin, reducing oxidative stress, inhibiting prostaglandin synthesis, and modulating vasodilation, as well as immunomodulatory actions in response to infections, trauma, and autoimmune diseases [27,28,29,30,31,32,33,34]. Hp is increased in parasitic, viral, and bacterial infections, as well as in diabetes, obesity cardiovascular disease, and peripheral vascular disease [35,36,37,38]. Hp levels were statistically significant in sixty patients injured in traffic accidents and were found to be a good indicator of the extent of brain damage in patients with severe head injuries [19,39].

Not all phenotypes behave similarly. The Hp1-1 phenotype is the most effective in suppressing inflammatory responses, while Hp2-2 predicts poor resolution and outcomes in coronary atherosclerosis and peripheral vascular disease [40]. In 2016, Hp2-2 was found to be an independent predictor of CSW after subarachnoid hemorrhage [41].

Hpr can be extruded from the cell with its signal peptide (HPRpsp) or without it (HPRwsp). Signal peptides or “transient peptides” are short peptides on the ends of newly synthesized proteins allowing the protein to exit the cell. Most often the new protein is extruded with its signal peptide, but other new proteins can be extruded without the signal peptide via “unconventional protein secretion” (UPS) [42,43].

CSW is a SIADH lookalike and has been described most commonly in patients with subarachnoid hemorrhage (SAH) [44,45], who present with salt wasting, volume depletion and volume-mediated appropriate induction of ADH secretion. Laboratory analysis reveals the same chemical profile in patients with classic ectopic SIADH and patients with IL-6-induced hyponatremia, both of whom are euvolemic with inappropriate ADH secretion. Volume depletion in the CSW patient is allegedly due to the presence of an acute phase reactant with natriuretic properties, identified as haptoglobin-related protein without signal peptide (HPRwsp) [7]. The authors studied the sera of a patient with SAH and another with Alzheimer disease, which induced brisk natriuresis when injected into rats. The sera were subjected to sequential windowed acquisition of all theoretical mass spectra (SWATH-MS) analysis, which identified increased levels of Hpr without signal peptide (Hpr-sp). Commercial recombinant HPRwsp also induced robust natriuresis in rats, but recombinant HPRpsp had no natriuretic effect. It is unknown why some infections or inflammatory states cause hyponatremia, and it could be surmised that genetic mutations regarding antigens, cytokine activation, and acute phase reactants are playing a role.

## 6. Reset Osmostat

RO is a form of hypotonic hyponatremia once described as a sub-type of SIADH or “type C-SIADH”. It is not due to inappropriate secretion of ADH but due to a “resetting” of the osmostat, the regulatory area in the hypothalamus which controls ADH secretion in response to hyperosmolarity or hypoosmolality. This reset osmostat controls ADH secretion appropriately but at a different, lower set point of serum osmolality between 280 and 290 mOsm/Kg [46]. If plasma osmolality increases, ADH secretion increases, and ADH is suppressed if the plasma osmolality decreases. Patients with RO present with a stable “reset point of osmolality”, maintaining a stable, low serum sodium, being able to dilute their urine if fluid-loaded or able to increase urine osmolality if fluid depleted. Conversely, the reset osmostat may be set at a higher plasma osmolality, a syndrome known as essential hypernatremia. These patients also respond normally to changes in plasma osmolality, maintaining serum sodium at a stable higher level.

Patients with RO were previously thought to be asymptomatic, but even mild hyponatremia can cause subtle neurological symptoms, including unsteady gait, imbalance, and falls [47]. Management involves serum sodium monitoring, adequate solute intake (salt), and moderate fluid restriction. Vaptan-like medications can cause a rapid overcorrection of serum sodium and should be used cautiously. RO is not inappropriate, appropriate, or autonomous secretion of ADH. It is a “resetting” of a normal response to changes in plasma osmolality.

## 7. Fractional Excretion of Urate—A Puzzling Phenomenon

A puzzling phenomenon that occurs in patients with forms of SIADH relates to the excretion of urate, measured as the fractional excretion of urate (FEurate) [48]. However, the measure of FEurate is only helpful after resolution of hyponatremia and is not to be used as a diagnostic tool. After correction of hyponatremia, it may indicate which form of hyponatremia was prevailing. The fractional excretion of uric acid as urate is the percent of filtered urate that is excreted in the urine. FEurate is elevated in patients with inappropriate ADH secretion (ectopic and IL-6-mediated) and also in patients with appropriate ADH secretion (CSW). Interestingly, the FEurate corrects to normal when the serum sodium is corrected in the inappropriate forms (ectopic and IL-6-mediated) but does not correct after correction of sodium in the appropriate form (CSW) [49]. It is surmised that the natriuretic peptide in CSW also interrupts urate reabsorption. Since urate reabsorption is uniquely relegated to the proximal renal tubule (PCT), it is likely that the natriuretic peptide also acts in the PCT. Non-correction of FEurate after correction of serum sodium in the CSW patient suggests that the acute phase protein, HPRwsp, is still present, and inflammation is ongoing [50]. More research is needed to show if HPRwsp is indeed a reliable biomarker to distinguish SIADH from CSW.

It is unknown why FEurate corrects after correction of serum sodium in patients with inappropriate ADH secretion (ectopic and IL-6-mediated). Some have suggested that PCT cells adapt to hypoosmolality by decreasing the intracellular solute content of diverse anions, resulting in decreased reabsorption of urate while the patient is hyponatremic [51]. The natriuresis that occurs in CSW has alternatively been ascribed to brain natriuretic protein (BNP) and atrial natriuretic protein (ANP). However, it is unlikely that BNP and ANP are the primary inducers of salt wasting in CSW, because patients with CSW (as well as patients with ectopic SIADH and IL-6-mediated SIADH) have increased FEurate, a solute which is exclusively reabsorbed in the PCT, a region not affected by BNP or ANP [52,53]. Nonetheless, levels of BNP and ANP are clearly increased in patients with CSW but are not inducers of natriuresis or uricosuria; rather, they are an epiphenomenon induced by inflammatory cytokines [54,55,56].

Additionally, it has been suggested that CSW is due to disruption of the neural input to the kidneys, with suppression of renin, angiotensin, and aldosterone, causing decreased PCT reabsorption of sodium and urate. However, articles on renal denervation for controlling hypertension do not describe urate excretion or potassium wasting [57]. Parenthetically, the levels of renin and aldosterone are elevated in volume-depleted CSW patients [58].

## 8. Summary

Classic, ectopic SIADH, IL-6-mediated SIADH, and CSW are three distinct syndromes resulting in hyponatremia and strikingly similar laboratory findings such as low Posm, inappropriately high Uosm, high Una, hypouricemia, and the absence of edema, with normal renal, adrenal, thyroid, and cardiac function. Patients with classic ectopic SIADH and IL-6-mediated SIADH are euvolemic, while patients with CSW are hypovolemic [59].

Misra et al. suggested simple bedside criteria to differentiate SIADH from CSW. SIADH patients should have (1) no evidence of hypovolemia, no hypotension, no postural hypotension, no dry mucus membranes, or tachycardia; (2) no lab evidence of dehydration, with normal hematocrit, albumin, and urea; (3) positive fluid balance with no weight loss; and (4) central venous pressure (CVP) > 6 cm water [60]. The lack of an easily available method to measure blood volume creates uncertainty in the diagnosis. However, current treatment recommendations allow a safe approach to patients without a firm pre-treatment knowledge of the pathogenetic mechanism involved.

## 9. Treatment of SIADH Overlaps with Treatment of CSW

The risks of profound hyponatremia include seizure, coma, and even death. Recognizing the rabbit hole of blindly treating hyponatremia, a balanced approach is recommended. Experts recommend a “stay the course” approach for the treatment of hyponatremia which is consistent with international guidelines. The European Clinical Practice Guidelines (ECPGs) recommend that correction of hyponatremia be limited to 10 mmol/L in the first day of treatment and 8 mmol/L for every subsequent day [61].

The US/Irish expert panel agreed with this approach, with a few added nuances regarding chronically hyponatremic patients with serum sodium < 120 mmol/L and > 48 h duration who were at normal risk of osmotic demyelination syndrome (ODS), recommending a correction limit of 10–12 mmol/L in any 12 h period and a minimum correction of 4–8 mmol/L. They also recommended increased attention for patients with sodium > 120 mmol/L at higher risk of ODS—namely patients with alcohol use disorder, hypokalemia, malnutrition, or advanced liver disease, in whom correction should not exceed 8 mmol/L in any 24 h period, and the minimum daily correction goal should be 4–6 mmol/L [62,63].

For patients with severe symptoms, the ECPG recommends bolus infusions of hypertonic saline in an effort to raise serum sodium by 5 mmol/L (European) or by 4–6 mmol/L (US/Irish panel) within a few hours. An increment of this magnitude is sufficient to markedly reduce intracranial pressure and reverse impending brain herniation [64,65]. These changes in practice guidelines have made ODS less common over the last few decades. Physicians are advised that correction of serum sodium < 120 mmol/L by greater than 10 mmol/L within 24 h should be avoided, because it can cause ODS. Rapidly correcting serum sodium levels during saline infusion should serve as an alert that the patient has CSW. Volume replacement in these patients will quickly inhibit the volume-mediated stimulus for ADH. Overly rapid correction of plasma sodium should be treated with urea or parenteral desmopressin.

In fact, urea may be used to slow the correction of serum sodium or as the initial treatment for hyponatremia. It is an “ineffective osmole”, and urea transporters allow diffusion across most cell membranes, but with limited access across the blood–brain barrier. As urea increases plasma osmolality, the osmotic gradient favors movement of water out of swollen brain cells. Over time the urea is excreted in the urine, along with electrolyte-free water, with urea being the solute. The loss of electrolyte-free water will increase serum sodium. As the urea gradient across the blood–brain barrier diminishes, the increase in serum sodium resulting from excretion of electrolyte-free water will prevent plasma water from re-entering the brain cells. In 2014, oral urea was recommended for the treatment of hyponatremia by the European Renal Association, the European Dialysis and Transplant Association, and the European Society of Intensive Care, although evidence for its use was weak. However, a systematic review of the literature by Wendt et al. concluded that urea was well tolerated, with a very low risk of overcorrection [66].

Desmopressin has been used to prevent a rapid correction of serum sodium in severe hyponatremia. Expert opinion recommends that serum sodium not be increased by more than 10–12 mEq/L in any 24 h period and/or 18 mEq/L in any 48-h period. Overcorrection is common if the patient has unexpected water diuresis, often in the patient with compulsive water drinking. Desmopressin combined with 3% saline appears to be a valid strategy to avoid unplanned overcorrection of hyponatremia [67,68].

Additional measures to reverse inflammation may require surgery, antibiotics, or corticosteroids, depending on which syndrome prevails.

## Figures and Tables

**Table 1:** Disorders causing SIADH.

**Nervous system:** acute psychosis, brain abscess, cavernous sinus thrombosis, cerebellar and cerebral atrophy, cerebrovascular accident, CNS lupus, delirium tremens, encephalitis (viral or bacterial), epilepsy, head trauma, herpes zoster, hydrocephalus, hypoxic ischemic encephalopathy, meningitis (viral, bacterial, tuberculosis, fungal), sub-arachnoid hemorrhage, subdural hematoma, Wernicke encephalopathy **Neoplastic disorders:** pulmonary (lung cancer, mesothelioma), gastrointestinal (carcinomas of the duodenum, pancreas, colon), genitourinary (adrenocortical carcinoma, carcinoma of cervix, ureter, bladder, prostate, ovary), other tumors (brain, carcinoid, leukemia, lymphoma) **Infections:** many bacterial/viral/fungal/mycoplasma infections, COVID-19 infection **Pulmonary disorders:** acute bronchitis, bronchiolitis, acute respiratory failure, asthma, atelectasis pneumothorax, empyema, emphysema, positive pressure ventilation, pulmonary fibrosis **Drugs:** arginine vasopressin (AVP) analogs [desmopressin, oxytocin]; drugs that stimulate release of ADH [vincristine, ifosfamide]; drugs that stimulate the vasopressin V2 receptor in the collecting duct [cisplatin, cyclophosphamide, chlorpropamide, selective serotonin reuptake inhibitors, carbamazepine, haloperidol, sertraline], thiazide diuretics, and other drugs **Other:** sarcoidosis, Shy–Drager syndrome, Guillain–Barre syndrome, acute intermittent porphyria, giant cell arteritis, multiple sclerosis, lupus erythematosus, post-operatively after general anesthesia **Hereditary:** upregulation of the V2- eceptor **Hormonal disorders:** hypothyroidism, hypopituitarism

*Adapted and modified from Cleveland Clinic website and SIADH Overview by Christie P. Thomas.*
